# Dynamic changes of serum vascular endothelial growth factor after surgical resection of hepatocellular carcinoma and its application in recurrence monitoring

**DOI:** 10.3389/fonc.2026.1865004

**Published:** 2026-10-05

**Authors:** Jirachaya Choovuthayakorn, Phichayut Phinyo, Noraworn Jirattikanwong, Peraphan Pothacharoen, Kanya Udomsin, Anon Chotirosniramit, Worakitti Lapisatepun, Sunhawit Junrungsee

**Affiliations:** 1 Division of Hepatobiliary and Pancreatic Surgery, Faculty of Medicine, Chiang Mai University, Chiang Mai, Thailand; 2 Department of Biomedical Informatics and Clinical Epidemiology (BioCE), Faculty of Medicine, Chiang Mai University, Chiang Mai, Thailand; 3 Thailand Excellence Center for Tissue Engineering and Stem Cell, Department of Biochemistry, Faculty of Medicine, Chiang Mai University, Chiang Mai, Thailand

**Keywords:** AFP (alpha-fetoprotein), dynamic change, HCC, recurrence, VEGF - vascular endothelial growth factor, hepatectomy

## Abstract

**Background:**

Hepatocellular carcinoma (HCC) recurs frequently after resection. Vascular endothelial growth factor (VEGF) mediates tumor angiogenesis, but its post-operative behavior is poorly described. We compared serum VEGF and alpha-fetoprotein (AFP) trajectories over the first post-operative year.

**Methodology:**

In this single-center prospective pilot study, 21 patients undergoing curative resection were assessed and 19 analyzed (8 recurrence, 11 non-recurrence). Both markers were measured before surgery and at 3, 6 and 12 months. Trajectories were compared using mixed-effects models with a group × time interaction (time categorical) and a random intercept; VEGF was modeled on the native scale, AFP on the log_10_ scale. All analyses are exploratory.

**Results:**

Eight of 19 patients (42.1%) recurred, at a median of 177.5 days. VEGF trajectories differed between groups (interaction p = 0.041). In the recurrence group, estimated mean VEGF fell from 188.4 to 82.5 pg/mL by 3 months, rose to 103.9 at 6 months and fell to 67.9 at 12 months. Reductions from baseline were significant in the recurrence group only (all p ≤ 0.003). The 6-month rise was not itself significant (+21.2 pg/mL; p = 0.552). Observed VEGF never differed between groups; AFP was higher in the recurrence group at 12 months (15.61 versus 2.04 IU/mL; p = 0.016).

**Conclusion:**

Serum VEGF declines markedly after resection, most steeply in patients who recur, and the groups follow different trajectories. The transient six-month rise coincided with the median time to recurrence but was not significant. Given the small, stage-imbalanced cohort, these findings are hypothesis-generating.

## Introduction

Hepatocellular carcinoma (HCC) is one of the most concerning cancers worldwide due to its high incidence and mortality. Moreover, these rates are expected to progressively increase over time. Though resection has shown to have an advantageous impact on the survival rate of patients with HCC in early Barcelona Clinic Liver Cancer (BCLC) staging (1), the main obstacle faced by many clinicians is the frequent recurrence after tumor removal. The recurrent rate of HCC was as great as 50 to 80 percent (2, 3). Various markers such as alpha-fetoprotein (AFP), prothrombin induced by vitamin K absence II (PIVKA II), glypican-3 (GPC3), and circulating tumor cells (CTC) are investigated and have shown the ability to detect recurrence (4–8). For the most widely used and accessible biomarker, AFP, though evidence has shown that high AFP levels in HCC patients correlate with poorer prognosis, the sensitivity of AFP for detecting recurrence of HCC is still 42 – 77% (9, 10). For emerging biomarkers, such as GPC-3, AFP L3, and CTC, although the sensitivity and specificity have improved, the laboratory methods and cost are complex and expensive (8, 11). Therefore, the affordable and available biomarker with more sensitivity and specificity is still the solution that can improve the prognosis of resected patients.

Vascular endothelial growth factor (VEGF) and its signaling pathway have long shown to be highly linked with the pathophysiology of HCC. This is due to its function in promoting new blood vessel formation, which is relevant to tumor growth and metastasis (12–14). The correlation of VEGF and HCC was also strongly demonstrated by the IMBRAVE150 trial (15), which showed the benefit of combining anti-VEGF and anti-programmed death-ligand 1 (PD-L1) in unresectable HCC. Even the prognostic value of pre-operative serum VEGF level has been intensively researched (4, 13, 16–21), but the data about how serum VEGF level changes after surgical resection is still scant. Based on the simple laboratory methods of detecting VEGF level, the value of VEGF dynamic changes as a tool for longitudinal monitoring should be explored. This study aims to investigate the patterns of changing serum VEGF levels in HCC patients who are undergoing surgical resection and to evaluate the efficacy of VEGF as a biomarker for recurrence monitoring.

## Methodology

### Study design

This study was a single-center, prospective observational pilot study conducted at the Division of Hepatobiliary and Pancreatic Surgery, Chiang Mai University Hospital, Thailand. The study was approved by the Research Ethics Committee of the Faculty of Medicine, Chiang Mai University (approval number FAC-MED-2564-08175) and was conducted in accordance with the principles of the Declaration of Helsinki.

Patients undergoing resection from June 2021 onwards were enrolled prospectively, and written informed consent was obtained from each before surgery. Clinical data and stored serum samples from seven patients resected between October 2020 and May 2021, six of whom met the criteria for the analysis set, were reviewed retrospectively under the same approval; for these patients, written consent for research use of stored specimens had been obtained at the time of collection. Enrollment closed in January 2022 and the database was closed on 31 December 2022.

### Participants

Consecutive patients diagnosed with hepatocellular carcinoma (HCC) who underwent liver resection with curative intent at our institution during the study period were eligible. The diagnosis was established by a hepatobiliary multidisciplinary team on the basis of contrast-enhanced magnetic resonance imaging or computed tomography, and was confirmed histologically in the resection specimen.

Patients were excluded if no post-operative serum sample could be obtained, or if follow-up was insufficient to determine recurrence status.

Twenty-one consecutive patients were assessed for eligibility and two were excluded: one died in hospital on post-operative day 27 from surgical complications before any post-operative sample could be obtained, and one was lost to follow-up at 10 months, so that recurrence status could not be determined. Nineteen patients were analyzed, 8 in the recurrence group and 11 in the non-recurrence group (**Figure 1**).

**Figure 1:**
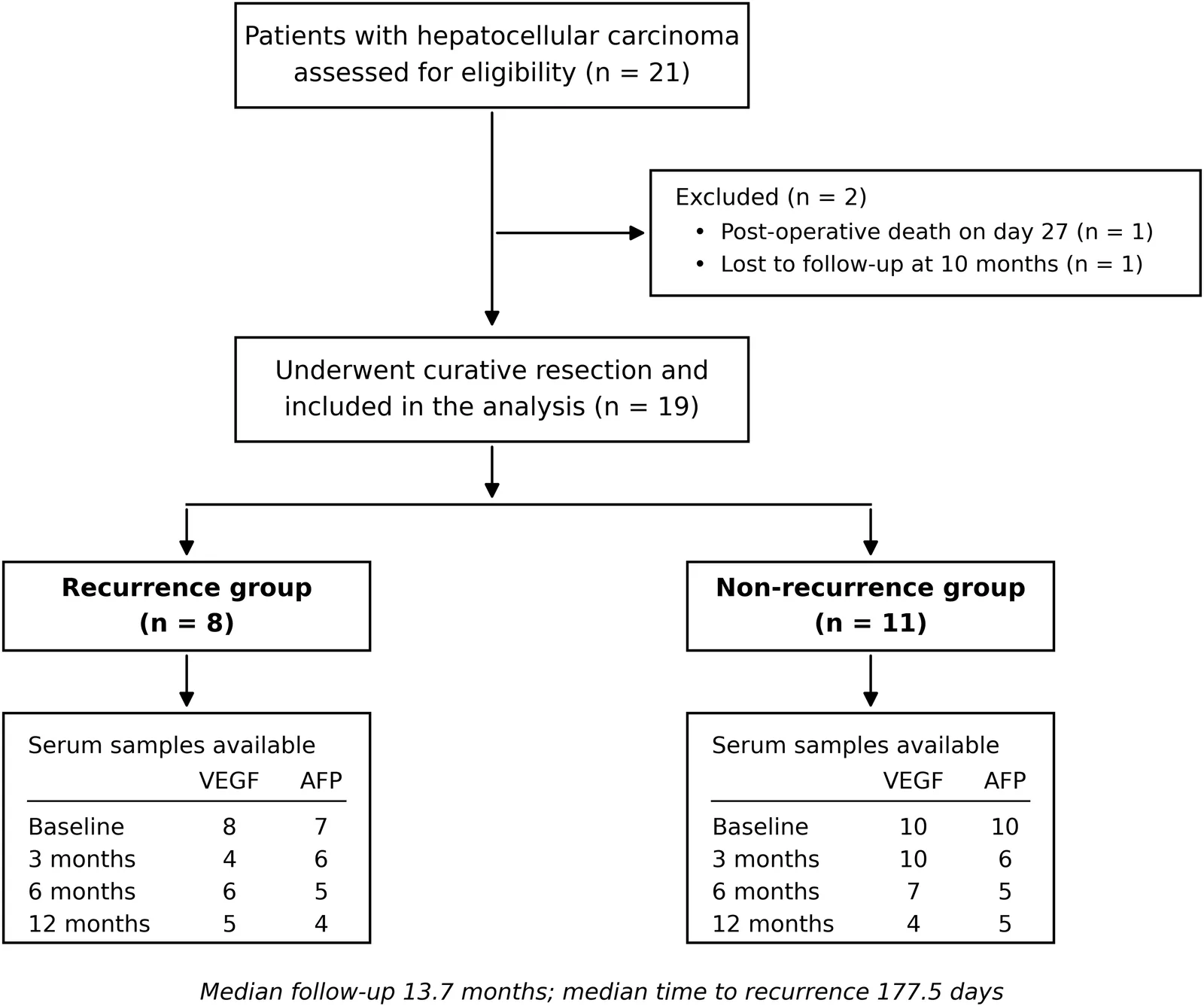
Flow of participants: 21 assessed for eligibility, 2 excluded (1 post-operative death on day 27; 1 lost to follow-up at 10 months), 19 analyzed (8 recurrence, 11 non-recurrence), with the number of available biomarker samples at each timepoint.

Three of the 19 analyzed patients (2 in the recurrence group, 1 in the non-recurrence group) had received transarterial chemoembolisation before resection. Because this intervention may raise circulating VEGF, these patients were retained in the primary analysis and a sensitivity analysis excluding them is reported.

### Definitions

Curative resection was defined as complete macroscopic removal of all identifiable tumor with a histologically negative resection margin (R0) on examination of the specimen.

Recurrence was defined as the appearance of a new intrahepatic or extrahepatic lesion with imaging characteristics diagnostic of HCC on contrast-enhanced computed tomography or magnetic resonance imaging, assessed by the hepatobiliary multidisciplinary team according to institutional consensus. Histological confirmation was obtained when a biopsy was clinically indicated. Surveillance imaging was performed at 3, 6 and 12 months after resection.

Group assignment. Patients were assigned to the recurrence group if recurrence was documented at any time during follow-up, and to the non-recurrence group otherwise. We note explicitly that this rule is not equivalent to recurrence status at 12 months: one patient developed recurrence at 17.7 months and is included in the recurrence group.

Disease-free survival was measured from the date of resection to the date of first recurrence or death from any cause, whichever occurred first, with patients who remained event-free censored at the date of last imaging. In this cohort no patient died without previously documented recurrence, so every disease-free survival event was a recurrence and death did not act as a competing event. One patient in the recurrence group died of HCC, 17 days after recurrence had been diagnosed.

Biomarker sampling relative to recurrence. For every serum sample we recorded whether it was obtained before or after the date on which recurrence was diagnosed, and a sensitivity analysis excluding post-diagnosis samples is reported.

### VEGF and AFP measurement

Serum was collected immediately before surgery and at 3, 6 and 12 months using plain serum tubes, centrifuged, and stored at −80 °C until analysis at the Department of Biochemistry, Faculty of Medicine, Chiang Mai University. Serum VEGF was measured in duplicate using the Quantikine ELISA Human VEGF Immunoassay (catalogue number DVE00; R&D Systems, Minneapolis, MN, USA), with a standard-curve range of 31.3–2000 pg/mL, intra-assay coefficient of variation 4.5–6.7% and inter-assay coefficient of variation 6.2–8.8%. Samples reading below the lowest standard were re-assayed undiluted, and the extrapolated value was retained. AFP was measured as part of routine clinical care.

### Statistical analysis

No formal sample-size calculation was performed. This was an exploratory single-center pilot study limited by the number of eligible patients undergoing resection during the enrollment period, and it was not powered to detect between-group differences at individual timepoints. All analyses are hypothesis-generating and all p-values are exploratory; no adjustment was made for multiplicity. The smallest detectable difference δ satisfies δ = (z_1−_α/_2_ + z_1−_β) × SD × √(1/n_1_ + 1/n_2_), with two-sided α = 0.05, 80% power and SD = 0.369, the pooled standard deviation of log_10_ baseline VEGF in this cohort; with n_1_ = 8 and n_2_ = 10, δ = 0.490 on the log_10_ scale, a 3.1-fold difference in geometric mean VEGF. Normally distributed continuous variables are presented as mean (standard deviation) and compared by independent-samples t-test; skewed variables as median (interquartile range) and compared by Mann–Whitney U test. Categorical variables are presented as n (%) and compared by Fisher’s exact test.

Biomarker trajectories were analyzed using linear mixed-effects models with fixed effects for group, time as a four-level categorical variable (0, 3, 6 and 12 months) and their interaction, and a subject-level random intercept, estimated by restricted maximum likelihood. Time was modeled categorically so that no functional form, in particular no linear or monotonic trend, was imposed on the trajectory. VEGF was modeled on the native scale, where residuals were compatible with normality (Shapiro–Wilk p = 0.132) and all estimates and intervals were positive. AFP was modeled on the log_10_ scale, because on the native scale its residuals departed markedly from normality (Shapiro–Wilk p = 1.1 × 10^-10^) and the model produced confidence intervals extending below zero, which a concentration cannot attain; AFP estimates are back-transformed and presented as geometric means with multiplicative confidence intervals.

Three contrasts were pre-specified: the group × time interaction; between-group differences at each timepoint; and, within the recurrence group, the 3- versus 6-month difference. For comparisons at individual timepoints, the comparison of observed values (Mann–Whitney U test, **Tables 1**, **2**) was regarded as the primary analysis and the corresponding model-based contrast as secondary. Sensitivity analyses comprised: modelling VEGF on the log_10_ scale; excluding samples drawn after recurrence was diagnosed; additional adjustment for BCLC stage (A versus B/C) and for platelet count; and excluding patients who had received pre-operative chemoembolisation. A landmark analysis restricted to patients disease-free at 3 months examined whether the change in VEGF from baseline to 3 months was associated with subsequent recurrence.

**Table 1:** Serum VEGF by group and timepoint, with number of available samples.

Timepoint	Recurrence, n	Recurrence, pg/mL, median (IQR)	Non-recurrence, n	Non-recurrence, pg/mL, median (IQR)	P
Pre-operative	8	180.45 (80.95–294.5)	10	91.8 (46.5–191.7)	0.096
3 months	4	94.3 (46.95–118.65)	10	81.1 (59.4–156.1)	0.945
6 months	6	89.4 (55.5–109.2)	7	39.0 (9.8–65.2)	0.181
12 months	5	64.2 (56.5–70.5)	4	78.15 (43.25–100.35)	0.286

IQR, interquartile range. Between-group comparisons by Mann–Whitney U test. Of the 22 missing VEGF observations, eleven occurred at attended visits at which AFP was successfully measured from the same blood draw, indicating loss of the serum aliquot or assay failure rather than loss to follow-up; two were not due, the patient having died; and nine had no sample available.

**Table 2:** Serum AFP by group and timepoint, with number of available samples.

Timepoint	Recurrence, n	Recurrence, IU/mL, median (IQR)	Non-recurrence, n	Non-recurrence, IU/mL, median (IQR)	P
Pre-operative	7	60.7 (7.36–24,583)	10	68.64 (1.87–831)	0.601
3 months	6	5.02 (1.6–24.3)	6	2.23 (1.5–4.3)	0.309
6 months	5	2.12 (1.38–4.27)	5	2.32 (1.38–2.41)	0.889
12 months	4	15.61 (8.67–163.5)	5	2.04 (1.06–2.26)	**0.016**

IQR, interquartile range. Between-group comparisons by Mann–Whitney U test.

The bold number are the p value that significance because they are less than 0.05.

The number of available samples at each timepoint is given in **Table 1**. The mixed-effects model assumes that missing observations are missing at random; eleven of the 22 missing VEGF observations occurred at visits the patient attended and at which AFP was successfully measured, indicating sample or assay loss rather than dropout.

Disease-free survival was estimated by the Kaplan–Meier method. Analyses were performed in Stata 17 (StataCorp, College Station, TX, USA); a two-sided p value below 0.05 was considered statistically significant. Figures were prepared using Python 3.12 with matplotlib.

## Results

### Baseline characteristics of the patients

Of 21 patients assessed for eligibility, 19 with HCC undergoing surgical resection were analyzed. Out of all patients, eight patients developed recurrence by the end of the study and 11 did not (**Figure 1**). The baseline characteristics are shown in **Table 3**. The mean age of patients in the recurrence group was slightly younger (52.3 ± 8.9 years) compared to the non-recurrence group (60.6 ± 6.2 years), a difference that was statistically significant (p = 0.028). Hemoglobin (11.29 ± 2.00 versus 14.01 ± 1.24 g/dL; p = 0.003) and albumin (3.81 ± 0.33 versus 4.45 ± 0.32 g/dL; p = 0.0009) were both significantly lower in the recurrence group. As can be expected, BCLC stage was more advanced in the recurrence group (stage A 3/8, B 4/8, C 1/8) than in the non-recurrence group, all of whom were stage A (p = 0.005). Alcohol consumption (6/8, 75.0% vs 0/10, 0%; p = 0.002) and smoking (4/8, 50.0% vs 0/10, 0%; p = 0.023) were more frequent in the recurrence group. There were no significant differences in other clinical parameters including sex, BMI, hepatitis profile, child-Pugh score, size and number of HCC, underlying diseases. The operative procedure did not differ between groups.

**Table 3:** Baseline characteristics of patients stratified by recurrence status.

Characteristic	Recurrence (n = 8)	Non-recurrence (n = 11)	P
Age, years, mean (SD)	52.3 (8.9)	60.6 (6.2)	0.028
Male sex, n (%)	6 (75.0)	7 (63.6)	1.000
Body mass index, kg/m², mean (SD)^a^	23.31 (3.46)	24.17 (3.90)	0.629
HBV infection, n (%)	4 (50.0)	6 (54.5)	1.000
HCV infection, n (%)	2 (25.0)	4 (36.4)	1.000
Child–Pugh grade A/B, n (%)	7 (87.5)/1 (12.5)	11 (100)/0 (0)	0.421
BCLC stage A/B/C, n (%)	3 (37.5)/4 (50.0)/1 (12.5)	11 (100)/0/0	**0.005**
Diabetes mellitus, n (%)	2 (25.0)	2 (18.2)	1.000
Hypertension, n (%)	2 (25.0)	8 (72.7)	0.070
Alcohol consumption, n (%)^a^	6 (75.0)	0 (0)	**0.002**
Smoking, n (%)^a^	4 (50.0)	0 (0)	**0.023**
Pre-operative chemoembolisation, n (%)	2 (25.0)	1 (9.1)	0.546
Hemoglobin, g/dL, mean (SD)^a^	11.29 (2.00)	14.01 (1.24)	**0.003**
Albumin, g/dL, mean (SD)^a^	3.81 (0.33)	4.45 (0.32)	**0.0009**
Total bilirubin, mg/dL, mean (SD)^a^	0.835 (0.72)	0.583 (0.34)	0.340
Platelet count, ×10^9^/L, mean (SD)^a^	227 (94)	221 (75)	0.884
INR, mean (SD)^a^	1.22 (0.21)	1.06 (0.12)	0.053
AFP, IU/mL, median (IQR)^b^	60.7 (7.36–24,583)	68.64 (1.87–831)	0.601
VEGF, pg/mL, median (IQR)^a^	180.45 (80.95–294.5)	91.8 (46.5–191.7)	0.096
Tumor size, cm, median (IQR)	5.8 (5.0–9.45)	4.8 (3.8–6.8)	0.300
Tumor number 1/2/3/>3, n (%)	6 (75.0)/0/0/2 (25.0)	9 (81.8)/1 (9.1)/1 (9.1)/0	0.230
Blood transfusion, n (%)^a^	3 (37.5)	1 (10.0)	0.275
Operation, n (%)			0.669
— Right or left hepatectomy	4 (50.0)	4 (36.4)	
— Sectionectomy	2 (25.0)	5 (45.5)	
— Segmentectomy	2 (25.0)	1 (9.1)	
— Wedge resection	0 (0)	1 (9.1)	

^a^ n = 10 in the non-recurrence group; one patient had no recorded value.

^b^ n = 7 (recurrence) and n = 10 (non-recurrence).

AFP, alpha-fetoprotein; BCLC, Barcelona Clinic Liver Cancer; HBV/HCV, hepatitis B/C virus; INR, international normalized ratio; IQR, interquartile range; SD, standard deviation; VEGF, vascular endothelial growth factor. Continuous variables compared by t-test or Mann–Whitney U test; categorical variables by Fisher’s exact test. No adjustment for multiplicity; p-values are exploratory.

The bold number are the p value that significance because they are less than 0.05.

Median follow-up, defined as the time from resection to recurrence, death or last contact, whichever occurred first, was 13.7 months (interquartile range 8.8–15.4). Total observation time ranged from 2.6 to 25.9 months, and the database was closed on 31 December 2022. Among patients who recurred, the median time to recurrence was 177.5 days (approximately 5.8 months). One patient in the recurrence group died of HCC. Median disease-free survival was 17.7 months, estimated at a point where only two patients remained at risk; the 12-month disease-free survival rate was 63.2%, with 11 patients at risk at that time (**Figure 2**).

**Figure 2:**
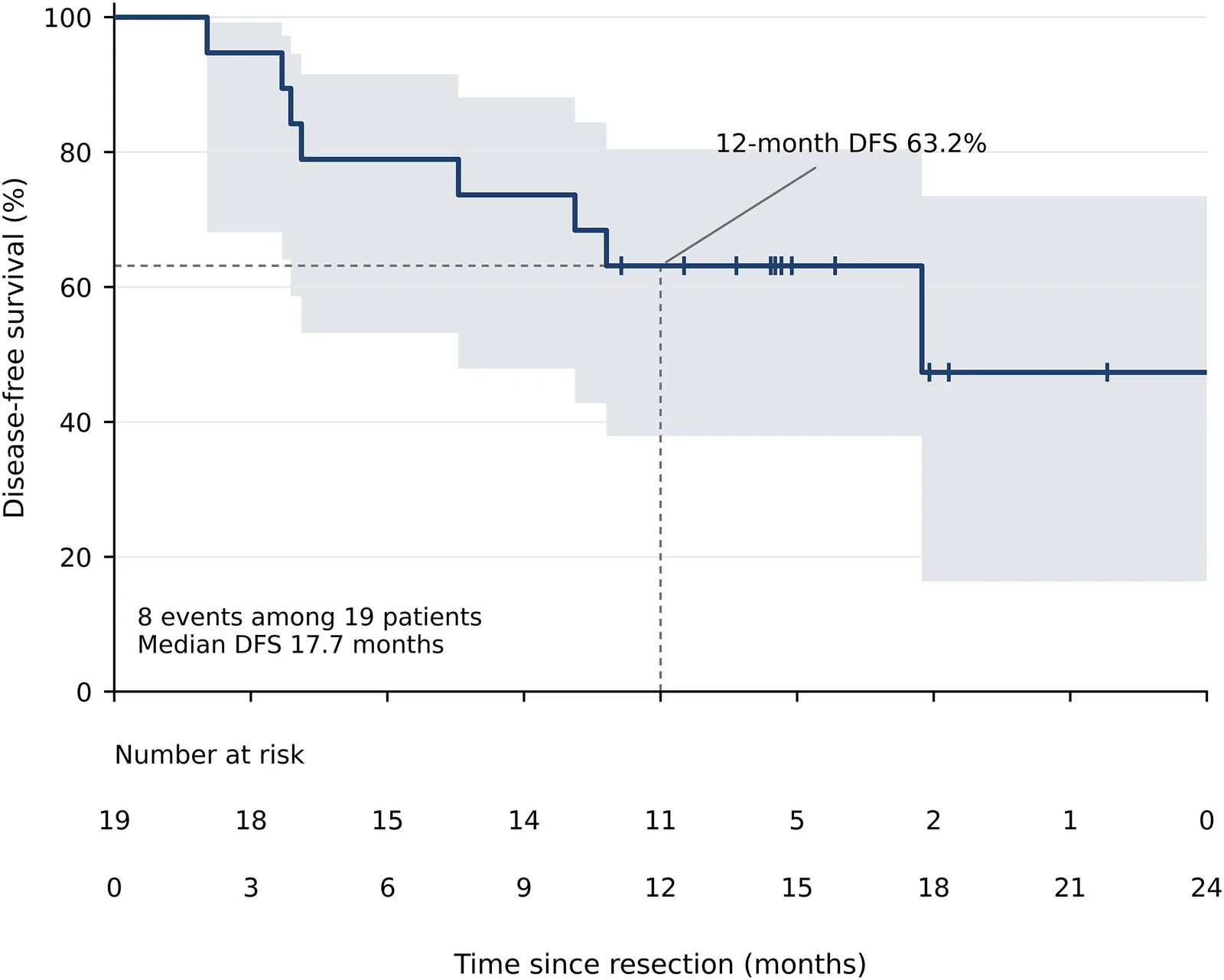
Kaplan–Meier estimate of disease-free survival for the 19 analyzed patients. Eight events were observed. The shaded band is the 95% confidence interval, calculated using Greenwood’s variance with a log–log transformation. Vertical ticks mark censored observations. The number of patients at risk is shown beneath the x-axis, beginning at 19. Few patients remained at risk beyond 15 months, so estimates in the right-hand portion of the curve are imprecise; the 12-month estimate was 63.2% and median disease-free survival was 17.7 months, reached when only two patients remained at risk.

### Changes in serum VEGF levels

Serum VEGF was measured at multiple time points: preoperative, 3 months, 6 months, and 12 months post-surgery (**Table 1**). Across the whole cohort, baseline serum VEGF had a median of 115.8 pg/mL (interquartile range 50.0–221.3; range 12.3–345.5; n = 18). Comparing the two groups, the median VEGF level in the recurrence group (180.5 pg/mL, IQR: 80.9-294.5) was slightly higher than those without recurrence (91.8 pg/mL, IQR: 46.5-191.7). However, this difference was not statistically significant (p = 0.096).

Serum VEGF fell after resection in both groups. In the recurrence group the observed median was 94.3 pg/mL (IQR 46.95–118.65) at 3 months, 89.4 pg/mL (55.5–109.2) at 6 months and 64.2 pg/mL (56.5–70.5) at 12 months. In the non-recurrence group the corresponding medians were 81.1 pg/mL (59.4–156.1), 39.0 pg/mL (9.8–65.2) and 78.15 pg/mL (43.25–100.35). None of the between-group differences at individual timepoints reached statistical significance (3 months p = 0.945; 6 months p = 0.181; 12 months p = 0.286). Trajectories are shown in **Figure 3A**.

**Figure 3:**
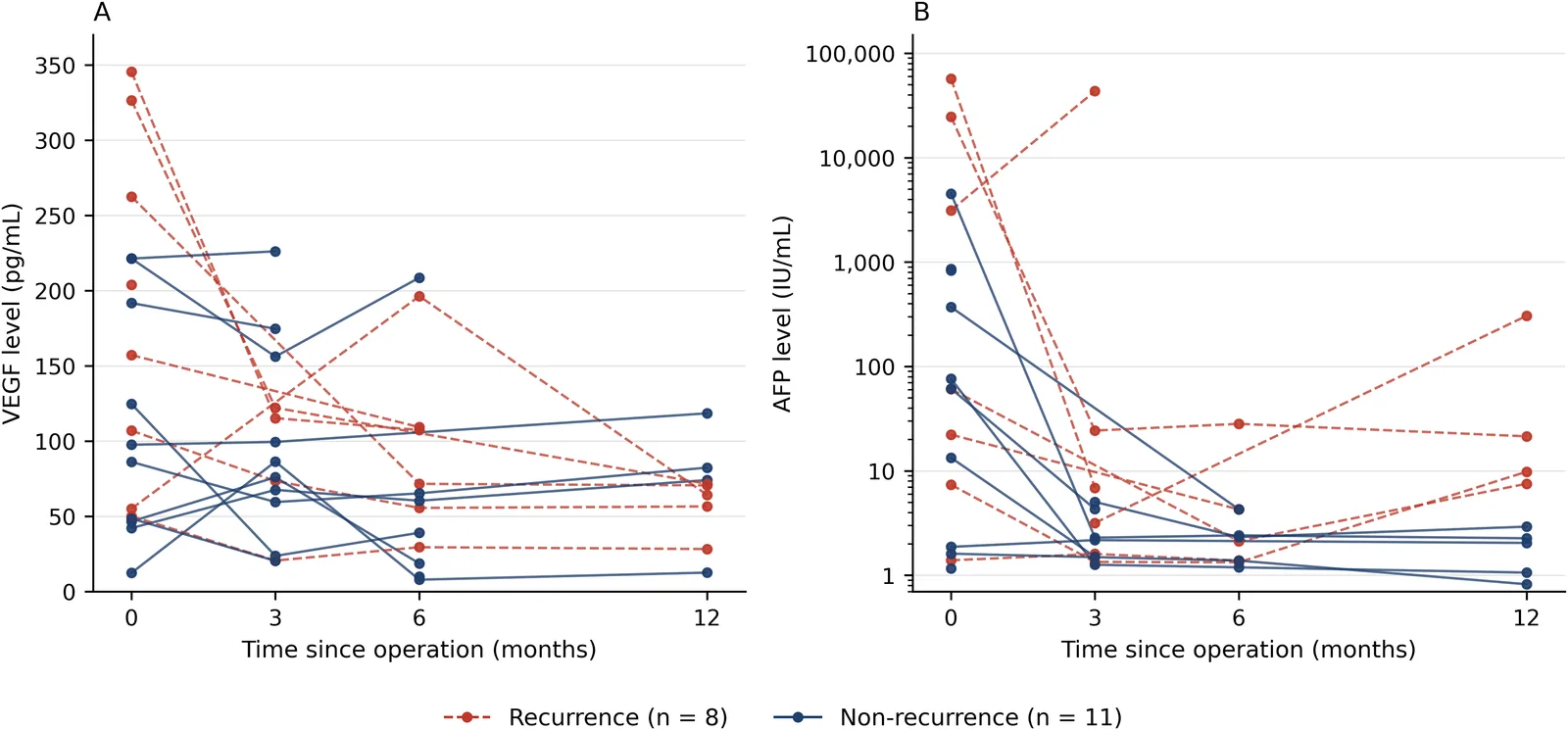
Individual patient trajectories of **(A)** serum VEGF (pg/mL) and **(B)** serum AFP (IU/mL) over 12 months after resection. Each line represents one patient; red dashed lines denote patients who developed recurrence and blue solid lines those who did not. The AFP axis is logarithmic. No fitted trend lines are shown.

### Changes in serum AFP levels

Serum AFP was measured at the same timepoints (**Table 2**; **Figure 3B**). Across the whole cohort, baseline serum AFP had a median of 61.25 IU/mL (interquartile range 7.36–860; range 1.16–57,020; n = 17). Baseline AFP was comparable between the recurrence group (median 60.7 IU/mL, IQR 7.36–24,583) and the non-recurrence group (68.64 IU/mL, IQR 1.87–831; p = 0.601).

AFP fell markedly after resection in both groups, to medians of 5.02 IU/mL (IQR 1.6–24.3) versus 2.23 IU/mL (1.5–4.3) at 3 months (p = 0.309), remaining low at 6 months (2.12 [1.38–4.27] vs 2.32 [1.38–2.41]; p = 0.889). At 12 months AFP rose again in the recurrence group to a median of 15.61 IU/mL (IQR 8.67–163.5), significantly higher than in the non-recurrence group (2.04 IU/mL, IQR 1.06–2.26; p **=** 0.016). This was the only statistically significant between-group difference observed for either marker at any individual timepoint.

### Modeled biomarker trajectories

Mixed-effects models were used to estimate trajectories while accommodating repeated measures and the unbalanced number of available observations (**Table 4**, **Figure 4**).

**Table 4:** Mixed-effects model estimates.

Panel A — VEGF, estimated mean (95% CI), pg/mL. Group × time interaction p = 0.041.				
Time	Recurrence	Non-recurrence	Between-group difference (95% CI)	p
Baseline	188.4 (140.5–236.3)	105.9 (63.6–148.2)	+82.4 (+13.8 to +151.0)	**0.019**
3 months	82.5 (21.4–143.7)	95.6 (53.3–137.9)	−13.2 (−93.3 to +67.0)	0.748
6 months	103.9 (51.1–156.8)	72.9 (25.2–120.6)	+31.3 (−45.5 to +108.0)	0.425
12 months	67.9 (11.5–124.3)	93.3 (34.3–152.1)	−25.2 (−113.3 to +63.0)	0.576
Panel B — VEGF, change from baseline (95% CI), pg/mL. Derived from the same model as Panel A.				
Time	Recurrence	p	Non-recurrence	p
3 months	−105.8 (−171.6 to −40.0)	**0.002**	−10.3 (−55.9 to +35.4)	0.660
6 months	−84.6 (−141.3 to −28.0)	**0.003**	−33.5 (−87.4 to +20.4)	0.223
12 months	−120.7 (−181.3 to −60.1)	**<0.001**	−13.1 (−77.6 to +51.4)	0.690
Panel C — AFP, estimated geometric mean (95% CI), IU/mL (log_10_ model). Group × time interaction p = 0.866.				
Time	Recurrence	Non-recurrence	Ratio, recurrence: non-recurrence (95% CI)	p
Baseline	256.2 (36.0–1,824.7)	56.8 (10.9–294.6)	4.51 (0.35–58.5)	0.249
3 months	13.8 (1.7–112.9)	2.57 (0.33–19.9)	5.38 (0.28–101.8)	0.262
6 months	8.71 (0.84–90.2)	1.85 (0.20–16.9)	4.70 (0.19–118.6)	0.347
12 months	38.0 (3.2–454.9)	2.01 (0.22–18.4)	18.97 (0.69–522.1)	0.082
Panel D — pre-specified contrasts				
Contrast		Estimate (95% CI)		p
VEGF, 3 → 6 months, recurrence group		+21.2 pg/mL (−48.6 to +91.0)		0.552
VEGF, 3 → 6 months, non-recurrence group		−23.2 pg/mL (−77.1 to +30.7)		0.398
VEGF, group × time interaction		—		**0.041**
AFP, group × time interaction		—		0.866
Panel E — sensitivity analyses for the VEGF group × time interaction				
Analysis	Observations (patients)	Group × time interaction, p	3 → 6 month change in the recurrence group (95% CI)	p
Primary model (VEGF, native scale)	54 (19)	**0.041**	+21.2 pg/mL (−48.6 to +91.0)	0.552
Adjusted for BCLC stage (A versus B/C)	54 (19)	**0.037**	+24.0 pg/mL (−46.5 to +94.6)	0.504
Adjusted for platelet count	50 (18)	0.075	+24.2 pg/mL (−49.0 to +97.3)	0.517
Excluding patients who had received pre-operative chemoembolisation	46 (16)	**0.029**	+29.8 pg/mL (−57.7 to +117.2)	0.505
Excluding samples drawn after the diagnosis of recurrence	43 (19)	**0.004**	−6.6 pg/mL (−75.6 to +62.5)	0.852
VEGF modeled on the log_10_ scale	54 (19)	0.179	× 1.19 (0.57 to 2.48)^a^	0.636

All models retain the structure of the primary analysis: fixed effects for group, time as a four-level categorical variable and their interaction, with a subject-level random intercept, estimated by restricted maximum likelihood. The interaction p value tests whether the two groups followed different trajectories; the final column tests the transient rise between 3 and 6 months within the recurrence group.

^a^ On the logarithmic scale the contrast is a fold-change (ratio of geometric means) rather than a difference in pg/mL.

Bold indicates p < 0.05. The analysis excluding samples drawn after the diagnosis of recurrence is restricted to the baseline, 3-month and 6-month timepoints, because every 12-month sample in the recurrence group was obtained after recurrence had been diagnosed. The platelet-adjusted model omits one patient for whom no platelet count was recorded. No adjustment was made for multiplicity; all p values are exploratory.

Because AFP is modeled on the logarithmic scale, between-group comparisons are expressed as ratios of geometric means (fold-differences) rather than as arithmetic differences. The 12-month comparison is p = 0.082 in this model but p = 0.016 by Mann–Whitney U test on the observed values (**Table 2**); the model is the more conservative of the two because the subject-level random intercept absorbs the very large between-patient variance in AFP.

**Figure 4:**
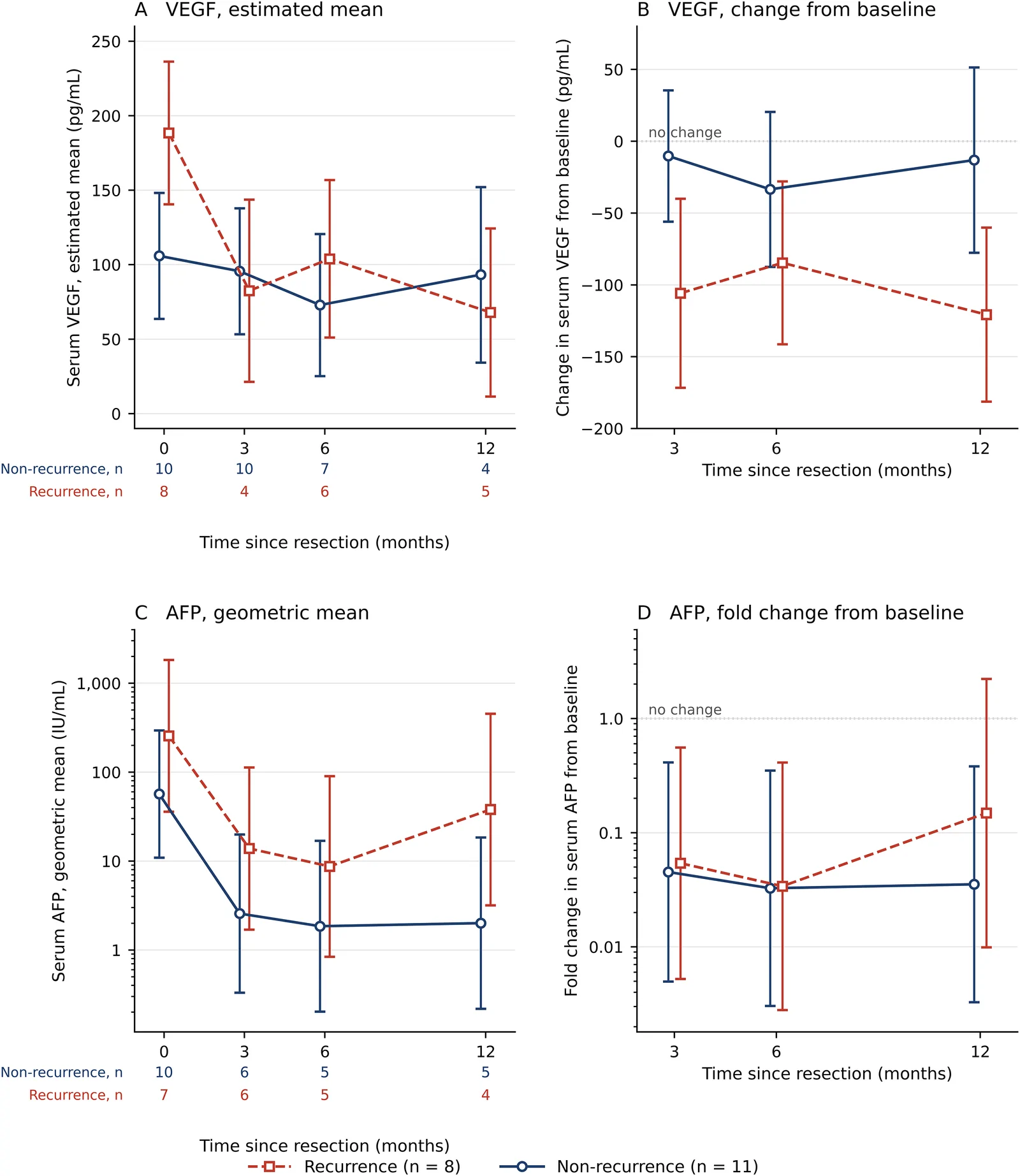
Mixed-model estimates for serum VEGF and serum AFP over the first post-operative year, by recurrence status. **(A)** Estimated mean serum VEGF at each timepoint. **(B)** Estimated change in serum VEGF from the pre-operative value; the dotted reference line at 0 indicates no change from baseline. **(C)** Estimated geometric mean serum AFP at each timepoint, plotted on a logarithmic axis. **(D)** Estimated fold change in serum AFP from the pre-operative value, plotted on a logarithmic axis; the dotted reference line at 1.0 indicates no change from baseline. Estimates are derived from linear mixed-effects models with fixed effects for group, time as a four-level categorical variable and their interaction, and a subject-level random intercept, fitted by restricted maximum likelihood. VEGF was modeled on the native scale; AFP was modeled on the log_10_ scale and the estimates in panels C and D are back-transformed, so that they are geometric means and ratios of geometric means respectively. Vertical bars are 95% confidence intervals. Points are offset horizontally to avoid overlap. The numbers below panels A and C are the observations contributing at each timepoint. Red squares with a dashed line, recurrence group (n = 8); blue circles with a solid line, non-recurrence group (n = 11). The group × time interaction was p = 0.041 for VEGF and p = 0.866 for AFP. AFP, alpha-fetoprotein; VEGF, vascular endothelial growth factor.

VEGF trajectories differed significantly between the two groups (group × time interaction, p = 0.041). In the recurrence group, estimated mean VEGF fell from 188.4 pg/mL (95% CI 140.5–236.3) before surgery to 82.5 pg/mL (21.4–143.7) at 3 months, rose to 103.9 pg/mL (51.1–156.8) at 6 months, and fell to 67.9 pg/mL (11.5–124.3) at 12 months. The non-recurrence group remained comparatively stable (105.9, 95.6, 72.9 and 93.3 pg/mL). Reductions from baseline were statistically significant at every timepoint in the recurrence group (−105.8, −84.6 and −120.7 pg/mL at 3, 6 and 12 months; all p ≤ 0.003) and at no timepoint in the non-recurrence group (all p > 0.2).

The rise between 3 and 6 months in the recurrence group coincided with the median time to recurrence of 177.5 days, but was not itself statistically significant (+21.2 pg/mL, 95% CI −48.6 to +91.0, p = 0.552). The between-group difference was significant at baseline (+82.4 pg/mL, 95% CI + 13.8 to +151.0, p = 0.019) but not at 6 months (+31.3 pg/mL, −45.5 to +108.0, p = 0.425).

For AFP, modeled on the log_10_ scale, the group × time interaction was not significant (p = 0.866). Estimated geometric mean AFP fell steeply from baseline in both groups and rose again at 12 months in the recurrence group only (**Table 4**).

### Sensitivity and landmark analyses

The interaction persisted after adjustment for BCLC stage (p = 0.037; **Table 4**, Panel E) and after excluding the three patients who had received pre-operative chemoembolisation (p = 0.029) and was stronger when samples drawn after the diagnosis of recurrence were excluded (p = 0.004). Adjustment for platelet count attenuated it to borderline significance (p = 0.075), and the interaction was not significant when VEGF was additionally log_10_-transformed (p = 0.179). The 3- to 6-month rise remained non-significant in every sensitivity analysis (p = 0.504 to 0.852).

In a landmark analysis restricted to the 14 patients who remained disease-free at 3 months, the fall in VEGF from baseline to 3 months was greater in those who subsequently recurred (median −60.8%, n = 4) than in those who did not (−3.5%, n = 10; p = 0.036). As simple discriminators of recurrence, baseline VEGF gave an area under the curve of 0.738 (p = 0.100) and baseline AFP 0.586 (p = 0.601).

## Discussion

The role of VEGF in the development of HCC is well established; increased expression in both tumor tissue and serum is linked to enhanced angiogenesis and increased tumor vascularity (4, 12, 14, 16, 18–22). The population in this study reflect the common baseline characteristics of HCC in Asian countries, where HBV infection predominates, and patients often present with large tumors. Notably, some patients at our center with BCLC stage B and C remained eligible for surgery. Our results demonstrated that the recurrence group tended to have higher baseline serum VEGF levels, which is associated with more advanced BCLC staging, a finding that parallels existing literature (23, 24), although the difference in observed values did not reach significance (p = 0.096).

One of the clearest findings in this study is a substantial fall in serum VEGF after resection, most marked in the recurrence group, who also had the highest pre-operative levels. Reductions from baseline were significant at all three post-operative timepoints in that group and at none in the non-recurrence group. There are two possible explanations for the lack of statistical significance in the non-recurrence group. First, the baseline VEGF in this group was relatively low; therefore, a statistically significant reduction may be harder to detect. Second, once the tumor is completely removed, VEGF levels are likely to stabilize, making a continuous longitudinal reduction unlikely. These findings contrast with the study by Hsiao et al. (20), which showed only a slight decrease in plasma VEGF at one and three years post-resection. Although the samples measured differed (serum vs. plasma), our study suggests that if measurements are performed frequently enough, a significant decrease in serum levels can be observed. Therefore, we believe that serum VEGF can serve as a monitoring tool once the primary source of production is removed, an effect that may be more pronounced in aggressive tumors. For early-stage or small tumors, however, the benefit of VEGF monitoring remains questionable.

The trajectories of the two groups also differed, as reflected in a significant group × time interaction (p = 0.041 on the native scale, p = 0.179 after log_10_ transformation). In the recurrence group, VEGF fell steeply to a nadir at 3 months and then rose at 6 months, producing a V-shaped configuration across the first half of the post-operative year, before falling again at 12 months. The 6-month rise coincided with the median time to recurrence of 177.5 days, raising the possibility that it reflects renewed angiogenic activity accompanying recurrent disease. This interpretation must remain provisional. The rise between 3 and 6 months did not reach statistical significance (+21.2 pg/mL, 95% CI −48.6 to +91.0, p = 0.552) and remained non-significant in every sensitivity analysis, and the significance of the interaction itself depends on the analysis scale. The pattern is therefore best regarded as an observation requiring confirmation in a larger cohort rather than as an established effect.

The two markers did not behave alike, although neither can be said to have outperformed the other. AFP was the only marker to differ significantly between the groups on the observed values at any individual timepoint (12 months, p = 0.016), although this difference did not reach significance in the mixed model (p = 0.082); observed VEGF did not differ significantly at any timepoint. Rather than indicating that one marker is superior, this suggests that the two carry different kinds of information. AFP behaved as a “spot check” that became informative once recurrent disease had had time to produce it, whereas VEGF changed earlier and more continuously in relation to tumor mass, so that its trajectory rather than any single value is the quantity of interest.

Interpretation is further constrained by substantial imbalance between the groups at baseline, which extended to BCLC stage, age, albumin, hemoglobin, alcohol use and smoking. All five patients with BCLC stage B or C disease recurred, compared with 3 of 13 with stage A, and no patient in the non-recurrence group reported alcohol use. With outcome perfectly separated by stage in one direction, no multivariable model containing these variables can be fitted, and the observed VEGF differences cannot be disentangled from tumor burden. Higher baseline VEGF in the recurrence group is at least as plausibly a marker of more advanced disease as an independent biological signal, and the stage-adjusted sensitivity analysis we present is unstable at this sample size and cannot resolve the question. This is a limitation of the cohort rather than of the analysis, and it is the principal reason we present these findings as hypothesis-generating.

The strength of this study lies in its being one of the few studies with paired pre-operative and multiple early post-operative measurements. Using a mixed-effects model, we demonstrated the trend, slope, and intercept of VEGF post-surgery, revealing a fluctuation in the recurrence group that was not observed with AFP.

The limitations of this study are the small sample size and the lack of other biomarkers, such as AFP L3 or PIVKA-II. Both groups are also strongly imbalanced and confounded by BCLC stage, as discussed above. The number of available samples differed by timepoint (**Table 1**), and the 6-month estimate on which the transient rise depends rests on six observations in the recurrence group, two of which were drawn after recurrence had been diagnosed. Pathological predictors of recurrence such as microvascular invasion, tumor grade, satellite nodules and background cirrhosis, were not available for adjustment. External validation of VEGF use for monitoring is also mandatory to support the findings in this study.

Taken together, the useful message of this study is narrow but real. Serum VEGF changes measurably and rapidly after resection of HCC, the trajectories of patients who do and do not recur differ, and this trajectory information is not captured by single AFP measurements. Whether monitoring the slope of serum VEGF adds anything to AFP and scheduled imaging is a question this study raises rather than answers. Testing it would require a larger, stage-matched cohort with sampling anchored to the date of recurrence rather than to the calendar, so that any change preceding radiological detection could be identified.

## Conclusion

Serum VEGF declines substantially after curative resection of HCC, most markedly in patients who subsequently develop recurrence, in whom pre-operative levels are also highest, and the trajectories of the two groups differ. A transient rise at six months coincided with the median time to recurrence but was not statistically significant. Because the cohort was small and strongly imbalanced for tumor stage, these findings are hypothesis-generating and do not establish clinical utility for recurrence monitoring. They support further evaluation of longitudinal VEGF trajectories, rather than single measurements, in an adequately powered and stage-matched study.

## Data Availability

The raw data supporting the conclusions of this article will be made available by the authors, without undue reservation.
