# Supplementary material for: Dynamic changes of serum vascular endothelial growth factor after surgical resection of hepatocellular carcinoma and its application in recurrence monitoring
Source: Front Oncol. 2026 Oct 5;16:1865004. doi: 10.3389/fonc.2026.1865004 (PMC13677535; doi:10.3389/fonc.2026.1865004)
Supplement: Supplementary file 1 [file DataSheet1.pdf]

STROBE Statement—checklist of items that should be included in reports of observational studies

Manuscript 1865004 — "Dynamic changes of serum vascular endothelial growth factor after surgical resection of hepatocellular carcinoma and its application in recurrence monitoring". Study design: single-centre prospective observational cohort (pilot). Page and paragraph numbers refer to the revised manuscript dated 20 August 2026.

	Item No.	Recommendation	Page No.	Relevant text from manuscript
**Title and abstract**				
**Title and abstract**	1	(a) Indicate the study's design with a commonly used term in the title or the abstract	Page 2, Abstract, Methodology	"In this single-center prospective pilot study, 21 patients undergoing curative resection were assessed and 19 analyzed (8 recurrence, 11 non-recurrence)."
		(b) Provide in the abstract an informative and balanced summary of what was done and what was found	Page 2, Paragraphs 1–4	Abstract (Background / Methodology / Results / Conclusion). The Conclusion states that, given the small, stage-imbalanced cohort, the findings are hypothesis-generating.
**Introduction**				
**Background/rationale**	2	Explain the scientific background and rationale for the investigation being reported	Page 3, Paragraphs 1–2	Introduction. Recurrence after resection, the limited sensitivity of AFP (42–77%), the role of VEGF in HCC angiogenesis, and the scarcity of data on post-operative change in serum VEGF.
**Objectives**	3	State specific objectives, including any prespecified hypotheses	Page 3, Paragraph 2 (final sentences)	"This study aims to investigate the patterns of changing serum VEGF levels in HCC patients who are undergoing surgical resection and to evaluate the efficacy of VEGF as a biomarker for recurrence monitoring." Three contrasts were pre-specified (Methods, Page 7).
**Methods**				
**Study design**	4	Present key elements of study design early in the paper	Page 4, Study design, Paragraph 1	"This study was a single-center, prospective observational pilot study conducted at the Division of Hepatobiliary and Pancreatic Surgery, Chiang Mai University Hospital, Thailand."
**Setting**	5	Describe the setting, locations, and relevant dates, including periods of recruitment, exposure, follow-up, and data collection	Page 4, Study design, Paragraphs 1–2	"Patients undergoing resection from June 2021 onwards were enrolled prospectively… Clinical data and stored serum samples from seven patients resected between October 2020 and May 2021, six of whom met the criteria for the analysis set, were reviewed retrospectively under the same approval… Enrollment closed in January 2022 and the database was closed on 31

				December 2022." Sampling at baseline and 3, 6 and 12 months (Page 6).
**Participants**	6	(a) Cohort study—Give the eligibility criteria, and the sources and methods of selection of participants. Describe methods of follow-up	Pages 4–5, Participants; Definitions	"Consecutive patients diagnosed with hepatocellular carcinoma (HCC) who underwent liver resection with curative intent at our institution during the study period were eligible… Patients were excluded if no post-operative serum sample could be obtained, or if follow-up was insufficient to determine recurrence status." Follow-up: "Surveillance imaging was performed at 3, 6 and 12 months after resection."
		(b) Cohort study—For matched studies, give matching criteria and number of exposed and unexposed	NA	This was not a matched cohort study. Groups were defined by recurrence status during follow-up, not by matching.
**Variables**	7	Clearly define all outcomes, exposures, predictors, potential confounders, and effect modifiers. Give diagnostic criteria, if applicable	Pages 5–6, Definitions; VEGF and AFP measurement	Definitions subsection defines curative resection (R0), recurrence (new intrahepatic or extrahepatic lesion with diagnostic imaging characteristics on contrast-enhanced CT or MRI, assessed by the multidisciplinary team), the group-assignment rule (recurrence at any time during follow-up), and disease-free survival. Exposure: serum VEGF and AFP. Potential confounders considered: BCLC stage, platelet count, pre-operative chemoembolisation.
**Data sources/ measurement**	8*	For each variable of interest, give sources of data and details of methods of assessment (measurement). Describe comparability of assessment methods if there is more than one group	Page 6, VEGF and AFP measurement	"Serum VEGF was measured in duplicate using the Quantikine ELISA Human VEGF Immunoassay (catalogue number DVE00; R&D Systems, Minneapolis, MN, USA), with a standard-curve range of 31.3–2000 pg/mL, intra-assay coefficient of variation 4.5–6.7% and inter-assay coefficient of variation 6.2–8.8%. Samples reading below the lowest standard were re-assayed undiluted, and the extrapolated value was retained. AFP was measured as part of routine clinical care." Identical methods were used in both groups; laboratory staff had no role in outcome assessment.
**Bias**	9	Describe any efforts to address potential sources of bias	Pages 5, 6, 7 and 13	Pre-operative chemoembolisation may raise circulating VEGF; these three patients were retained in the primary analysis and a sensitivity analysis excluding them is reported (Page 5). For every sample it was recorded whether it

				was obtained before or after the diagnosis of recurrence, and a sensitivity analysis excluding post-diagnosis samples is reported (Page 5). Missing data are accounted for in full (Page 7). The Limitations paragraph states that the 6-month estimate rests on six observations, two of which were drawn after recurrence had been diagnosed (Page 13).
**Study size**	10	Explain how the study size was arrived at	Page 6,<br>Statistical analysis,<br>Paragraph 1	"No formal sample-size calculation was performed. This was an exploratory single-center pilot study limited by the number of eligible patients undergoing resection during the enrollment period… The smallest detectable difference δ satisfies $\delta = (z_{1-\alpha/2} + z_{1-\beta}) \times SD \times \sqrt{(1/n_1 + 1/n_2)}$, with two-sided $\alpha = 0.05$, 80% power and SD = 0.369… with $n_1 = 8$ and $n_2 = 10$, $\delta = 0.490$ on the $\log_{10}$ scale, a 3.1-fold difference in geometric mean VEGF."
**Quantitative variables**	11	Explain how quantitative variables were handled in the analyses. If applicable, describe which groupings were chosen and why	Pages 6–7,<br>Statistical analysis	"Normally distributed continuous variables are presented as mean (standard deviation) and compared by independent-samples t-test; skewed variables as median (interquartile range) and compared by Mann–Whitney U test." "Time was modeled categorically so that no functional form, in particular no linear or monotonic trend, was imposed on the trajectory." VEGF was modelled on the native scale and AFP on the $\log_{10}$ scale, with the residual diagnostics given (Shapiro–Wilk $p = 0.132$ and $p = 1.1 \times 10^{-10}$).
**Statistical methods**	12	(a) Describe all statistical methods, including those used to control for confounding	Pages 6–7,<br>Statistical analysis	"Biomarker trajectories were analyzed using linear mixed-effects models with fixed effects for group, time as a four-level categorical variable (0, 3, 6 and 12 months) and their interaction, and a subject-level random intercept, estimated by restricted maximum likelihood." The primary model is unadjusted; confounding was explored by additional adjustment for BCLC stage (A versus B/C) and platelet count as sensitivity analyses. Analyses in Stata 17; figures in Python 3.12 with matplotlib.
		(b) Describe any methods used to examine subgroups and interactions	Page 7,<br>Paragraph 2	"Three contrasts were pre-specified: the group × time interaction; between-group

				differences at each timepoint; and, within the recurrence group, the 3- versus 6-month difference." The group × time interaction is the primary test of differing trajectories.
		(c) Explain how missing data were addressed	Page 7,<br>Paragraph 3;<br>Table 2 footnote	"The mixed-effects model assumes that missing observations are missing at random; eleven of the 22 missing VEGF observations occurred at visits the patient attended and at which AFP was successfully measured, indicating sample or assay loss rather than dropout." Table 2 footnote gives the full breakdown: eleven attended visits with AFP measured, two not due (patient having died), nine with no sample available. No imputation was performed; maximum likelihood uses all available observations.
		(d) Cohort study—If applicable, explain how loss to follow-up was addressed	Pages 4–5,<br>Participants;<br>Definitions	One of the 21 patients assessed was excluded because loss to follow-up at 10 months made recurrence status indeterminate. For disease-free survival, "patients who remained event-free [were] censored at the date of last imaging". No patient in the analysis set was lost to follow-up before the closure date.
		(e) Describe any sensitivity analyses	Page 7 (methods);<br>Page 10 (results);<br>Table 4, Panel E	"Sensitivity analyses comprised: modelling VEGF on the $\log_{10}$ scale; excluding samples drawn after recurrence was diagnosed; additional adjustment for BCLC stage (A versus B/C) and for platelet count; and excluding patients who had received pre-operative chemoembolisation. A landmark analysis restricted to patients disease-free at 3 months examined whether the change in VEGF from baseline to 3 months was associated with subsequent recurrence."
**Results**				
**Participants**	13*	(a) Report numbers of individuals at each stage of study—eg numbers potentially eligible, examined for eligibility, confirmed eligible, included in the study, completing follow-up, and analysed	Pages 4–5;<br>Page 8,<br>Paragraph 1;<br>Figure 1	"Twenty-one consecutive patients were assessed for eligibility and two were excluded… Nineteen patients were analyzed, 8 in the recurrence group and 11 in the non-recurrence group (Figure 1)." Figure 1 also reports the number of biomarker samples available at each timepoint; Tables 2 and 3 give these per group.

		(b) Give reasons for non-participation at each stage	Pages 4–5; Figure 1	"one died in hospital on post-operative day 27 from surgical complications before any post-operative sample could be obtained, and one was lost to follow-up at 10 months, so that recurrence status could not be determined."
		(c) Consider use of a flow diagram	Figure 1 (Page 23, legend)	Figure 1 presents the flow of participants: 21 assessed, 2 excluded with reasons, 19 analysed (8 recurrence, 11 non-recurrence), with available samples at each timepoint.
**Descriptive data**	14*	(a) Give characteristics of study participants (eg demographic, clinical, social) and information on exposures and potential confounders	Page 8, Paragraph 1; Table 1 (Pages 16–17)	Table 1 reports age, sex, body mass index, hepatitis status, Child–Pugh grade, BCLC stage, comorbidity, alcohol use, smoking, pre-operative chemoembolisation, laboratory values, baseline AFP and VEGF, tumour size and number, blood transfusion and operation type, by group. Significant imbalances: age (p = 0.028), hemoglobin (p = 0.003), albumin (p = 0.0009), BCLC stage (p = 0.005), alcohol (p = 0.002), smoking (p = 0.023).
		(b) Indicate number of participants with missing data for each variable of interest	Page 7; Tables 1–3 and footnotes	Table 1 footnotes: "[a] n = 10 in the non-recurrence group; one patient had no recorded value. [b] n = 7 (recurrence) and n = 10 (non-recurrence)." Tables 2 and 3 give the number of contributing observations at each timepoint for each group (VEGF 8/4/6/5 and 10/10/7/4; AFP 7/6/5/4 and 10/6/5/5). The Table 2 footnote classifies all 22 missing VEGF observations.
		(c) Cohort study—Summarise follow-up time (eg, average and total amount)	Page 8, Paragraph 2	"Median follow-up, defined as the time from resection to recurrence, death or last contact, whichever occurred first, was 13.7 months (interquartile range 8.8–15.4). Total observation time ranged from 2.6 to 25.9 months, and the database was closed on 31 December 2022."
**Outcome data**	15*	Cohort study—Report numbers of outcome events or summary measures over time	Page 8, Paragraphs 1–2; Figure 2	"eight patients developed recurrence by the end of the study and 11 did not (Figure 1)… the median time to recurrence was 177.5 days (approximately 5.8 months). One patient in the recurrence group died of HCC. Median disease-free survival was 17.7 months… the 12-month disease-free survival rate was 63.2%, with 11 patients at risk at that time (Figure 2)."

**Main results**	16	(a) Give unadjusted estimates and, if applicable, confounder-adjusted estimates and their precision (eg, 95% confidence interval). Make clear which confounders were adjusted for and why they were included	Pages 9–10;<br>Table 4,<br>Panels A–D<br>(Pages 20–21)	All primary estimates are unadjusted and reported with 95% confidence intervals. "VEGF trajectories differed significantly between the two groups (group × time interaction, p = 0.041)… estimated mean VEGF fell from 188.4 pg/mL (95% CI 140.5–236.3)… to 82.5 pg/mL (21.4–143.7) at 3 months, rose to 103.9 pg/mL (51.1–156.8) at 6 months, and fell to 67.9 pg/mL (11.5–124.3) at 12 months." Confounder-adjusted estimates are presented only as sensitivity analyses (BCLC stage and platelet count, Table 4 Panel E), because outcome is perfectly separated by stage and no stable multivariable model can be fitted at this sample size (Discussion, Page 12).
		(b) Report category boundaries when continuous variables were categorized	Pages 6–7	Time was categorised at the four scheduled sampling points (pre-operative, 3, 6 and 12 months). BCLC stage was dichotomised for the sensitivity analysis as "A versus B/C". No biomarker value was categorised; both markers were analysed on a continuous scale.
		(c) If relevant, consider translating estimates of relative risk into absolute risk for a meaningful time period	NA	No relative-risk estimates were derived. The analysis compares mean and geometric-mean biomarker trajectories, and disease-free survival is reported as an absolute probability (12-month rate 63.2%).
**Other analyses**	17	Report other analyses done—eg analyses of subgroups and interactions, and sensitivity analyses	Page 10,<br>Sensitivity and landmark analyses;<br>Table 4, Panel E	Five sensitivity analyses (interaction p = 0.041 primary; 0.037 adjusted for BCLC stage; 0.075 adjusted for platelet count; 0.029 excluding pre-operative chemoembolisation; 0.004 excluding post-diagnosis samples; 0.179 with VEGF on the $\log_{10}$ scale). Landmark analysis in the 14 patients disease-free at 3 months (median fall −60.8% versus −3.5%, p = 0.036). Baseline VEGF and AFP as simple discriminators (AUC 0.738, p = 0.100; 0.586, p = 0.601).
**Discussion**				
**Key results**	18	Summarise key results with reference to study objectives	Pages 11–12,<br>Paragraphs 1–3	"One of the clearest findings in this study is a substantial fall in serum VEGF after resection, most marked in the recurrence group, who also had the highest pre-operative levels… The trajectories of the two groups also differed, as reflected in a significant

				group × time interaction ($p = 0.041$ on the native scale, $p = 0.179$ after $\log_{10}$ transformation)."
**Limitations**	19	Discuss limitations of the study, taking into account sources of potential bias or imprecision. Discuss both direction and magnitude of any potential bias	Pages 12–13	Baseline imbalance paragraph: "With outcome perfectly separated by stage in one direction, no multivariable model containing these variables can be fitted, and the observed VEGF differences cannot be disentangled from tumor burden. Higher baseline VEGF in the recurrence group is at least as plausibly a marker of more advanced disease as an independent biological signal." Limitations paragraph: small sample size; absence of AFP-L3 and PIVKA-II; unequal sample numbers by timepoint; the 6-month estimate resting on six observations, two drawn after diagnosis of recurrence; pathological predictors unavailable for adjustment.
**Interpretation**	20	Give a cautious overall interpretation of results considering objectives, limitations, multiplicity of analyses, results from similar studies, and other relevant evidence	Pages 12–13; Conclusion, Page 14	Link to objective: the trajectories differ, but "the significance of the interaction itself depends on the analysis scale. The pattern is therefore best regarded as an observation requiring confirmation in a larger cohort rather than as an established effect." Comparison with other studies: contrasted with Hsiao et al. (20). Multiplicity: "no adjustment was made for multiplicity; all p values are exploratory" (Page 6). Cautious interpretation: "these findings are hypothesis-generating and do not establish clinical utility for recurrence monitoring" (Conclusion).
**Generalisability**	21	Discuss the generalisability (external validity) of the study results	Pages 11 and 13	"The population in this study reflect the common baseline characteristics of HCC in Asian countries, where HBV infection predominates, and patients often present with large tumors. Notably, some patients at our center with BCLC stage B and C remained eligible for surgery." "External validation of VEGF use for monitoring is also mandatory to support the findings in this study." Testing would "require a larger, stage-matched cohort with sampling anchored to the date of recurrence rather than to the calendar".
**Other information**				
**Funding**	22	Give the source of funding and the role	Page 15,	"This research received no specific grant

		of the funders for the present study and, if applicable, for the original study on which the present article is based	Funding	from any funding agency in the public, commercial or not-for-profit sectors."

*Give information separately for cases and controls in case-control studies and, if applicable, for exposed and unexposed groups in cohort and cross-sectional studies.

Note: An Explanation and Elaboration article discusses each checklist item and gives methodological background and published examples of transparent reporting. The STROBE checklist is best used in conjunction with this article (freely available on the Web sites of PLoS Medicine at http://www.plosmedicine.org/, Annals of Internal Medicine at http://www.annals.org/, and Epidemiology at http://www.epidem.com/). Information on the STROBE Initiative is available at www.strobe-statement.org.
